# Olfactory bulb hypoplasia in Prokr2 null mice stems from defective neuronal progenitor migration and differentiation

**DOI:** 10.1111/j.1460-9568.2007.05958.x

**Published:** 2007-12

**Authors:** Haydn M Prosser, Allan Bradley, Maeve A Caldwell

**Affiliations:** 1 The Wellcome Trust Sanger Institute, Wellcome Trust Genome Campus Hinxton, Cambridge CB10 1SA, UK; 2 Centre for Brain Repair and Department of Clinical Neurosciences, University Forvie Site Robinson Way, Cambridge CB2 2PY2, UK

**Keywords:** neural progenitor, olfactory bulb, prokineticin receptor 2, rostral migratory stream, subventricular zone

## Abstract

New neurons are added on a daily basis to the olfactory bulb (OB) of a mammal, and this phenomenon exists throughout its lifetime. These new cells are born in the subventricular zone and migrate to the OB via the rostral migratory stream (RMS). To examine the role of the prokineticin receptor 2 (Prokr2) in neurogenesis, we created a Prokr2 null mouse, and report a decrease in the volume of its OB and also a decrease in the number of bromodeoxyuridine (BrdU)-positive cells. There is disrupted architecture of the OB, with the glomerular layer containing terminal dUTP nick-end labeling (TUNEL) -positive nuclei and also a decrease in tyrosine hydroxylase-positive neurons in this layer. In addition, there are increased numbers of doublecortin-positive neuroblasts in the RMS and increased PSA-NCAM (polysialylated form of the neural cell adhesion molecule) -positive neuronal progenitors around the olfactory ventricle, indicating their detachment from homotypic chains is compromised. Finally, in support of this, Prokr2-deficient cells expanded *in vitro* as neurospheres are incapable of migrating towards a source of recombinant human prokineticin 2 (PROK2). Together, these findings suggest an important role for Prokr2 in OB neurogenesis.

## Introduction

It is well established that there are two main areas where neurogenesis occurs in the adult mammalian brain, the dentate gyrus of the hippocampus and the olfactory bulb (OB; for reviews, see Gould *et al*., 1999; Alvarez-Buylla & Garcia-Verdugo, 2002; Watts *et al*., 2005). In the murine brain, the OB granular and periglomerular layers are supplied with new interneurons originating in the lateral wall of the subventricular zone (SVZ), which migrate there via the rostral migratory stream (RMS; Kaplan & Hinds, 1977; Luskin, 1993).

The prokineticins (Prok1 and Prok2) are two recently identified secreted bioactive molecules (Li *et al*., 2001; LeCouter *et al*., 2003). Signal transduction of both molecules is via two closely related G-protein-coupled receptors (Prokr1 and Prokr2; Lin *et al*., 2002; Masuda *et al*., 2002; Soga *et al*., 2002). To date several physiological roles have been attributed to the prokineticins and their receptors: most notably gut motility (Li *et al*., 2001), reproductive function (Matsumoto *et al*., 2006; Maldonado-Perez *et al*., 2007) and circadian output from the suprachiasmatic nuclei (Cheng *et al*., 2002; Li *et al*., 2006; Prosser *et al*., 2007). Expression analysis has revealed spatially distinct and overlapping areas of expression of prokineticins and their receptors (Li *et al*., 2001; Cheng *et al*., 2002; Lin *et al*., 2002; Masuda *et al*., 2002; Ng *et al*., 2005; Matsumoto *et al*., 2006). Of particular relevance to this study, Prokr2 and Prokr1 expression has been detected by *in situ* hybridization in the SVZ, RMS, olfactory ventricle ependyma and subependymal layers, while Prok2, but not Prok1, is expressed in the OB granular and perigranular layers (Ng *et al*., 2005; Matsumoto *et al*., 2006). More recently, the expression of both prokineticins and their receptors have been mapped by *in situ* hybridization in the adult mouse brain, demonstrating that Prok2 and Prokr2 are more widely expressed in the brain than Prok1 and Prokr1 (Cheng *et al*., 2006).

In this study we have generated a null mutant mouse line for Prokr2 (Prosser *et al*., 2007) that shows abnormal development of the OB in accordance with that previously described for the *Prokr2* (Matsumoto *et al*., 2006) and *Prok2* knockouts (Ng *et al*., 2005). We describe the fine-scale OB structural deformities in *Prokr2* null mice, and the effect on neuroblast migration from the SVZ to the OB. In addition, we describe a role for Prokr2 in chemoattraction within the OB and *in vitro*.

## Materials and methods

### Gene knockout

The *prokr2*^*Brdm1*^ allele (abbreviated here to *m*) was confirmed by radio-ligand binding assay to be a functional null (Prosser *et al*., 2007). The mice were of mixed 129:C57B/6 genetic background, and were F1 or F2 intercrosses of mice backcrossed four–six times to the C57B/6 genetic background. All studies were licensed by the UK Home Office under the Animals (Scientific Procedures) Act 1986.

### Bromodeoxyuridine (BrdU) administration

Mice either received BrdU (300 mg/kg i.p.) as a single injection at 6 weeks of age and were perfused (4% paraformaldehyde) 21 days later (genotypes: 7 *m/m*, 10 +/*m*, 6 +/+), or received five daily injections of BrdU (50 mg/kg) and then perfused 1 day later (genotypes: 8 *m/m*, 7 +/*m*, 7 +/+), or received a single injection of BrdU (100 mg/kg) and perfused 2 h later (genotypes: 3 *m/m*, 4 +/*m*, 3 +/+). Mice were given a lethal dose of Euthatal anaesthetic prior to perfusion. Brains were postfixed overnight and equilibrated in 30% sucrose.

### Immunohistochemistry

A 1 : 6 series of free-floating sections (40 µm) was immunostained as follows: sheep anti-BrdU (1 : 3000; Fitzgerald), mouse anti-tyrosine hydroxylase (TH; 1 : 500; Chemicon), goat anti-doublecortin (DCX; 1 : 400; Santa Cruz), rabbit anti-calbindin (1 : 500; Sigma) and the polysialylated form of the neural cell adhesion molecule (PSA-NCAM) (1 : 500; Chemicon). For diaminobenzidine (DAB) staining sections were treated with 10% hydrogen peroxide in 10% methanol (5 min), 2 N HCl at 37 °C (20 min), borate buffer (2 × 10 min), Tris-buffered saline (TBS) containing 0.2% Triton and 5% normal donkey serum (NDS; 2 h). Anti-sheep BrdU incubation was overnight at 4 °C followed by three × TBS washes. Secondary antibody [anti-sheep biotin (1 : 400) 2 h], followed by three × TBS washes and avidin-biotin complex (Vectastain ABC kit, Vector Laboratories) in TBS (2 h). Antigens were visualized using DAB. For TH, calbindin and DCX staining the HCl step was omitted and a secondary antibody against mouse, rabbit or goat, respectively, was used. For immunofluorescent staining the quenching and avidin-biotin complex steps were omitted. Anti-mouse IgM-biotin was used for PSA NCAM. The tertiary antibody used was strepavidin-Alexa-568. Hoescht was included in final antibody incubations as a nuclear stain. Terminal dUTP nick-end labeling (TUNEL) staining was carried out on 40-µm brain sections using Manufacturer's instructions (Roche).

### Transwell assay

SVZ was excised from N6F2 generation P0 pups under sterile conditions. Following accutase (Sigma) treatment (10 min) and three × washes with Dulbecoo's modified essential medium (DMEM)/B27 (2%), cells were triturated. Viable cells were counted and seeded at 200 K/mL in DMEM/HAMS F12 (3 : 1), B27 (2%), 100 units penicillin/100 mg streptomycin/0.25 mg amphotericin, fibroblast growth factor (FGF)-2 (20 ng/mL), epidermal growth factor (EGF; 20 ng/mL) and heparin (5 µg/mL). After 4 days, cells dissociated with accutase were plated at 25 K/mL onto the upper chamber of an 8-µm transwell (ChemoTx, Neuro Probe) previously coated with fibronectin (1 µg/mL). Lower chamber media was plus or minus recombinant PROK2 (30 ng/mL). After 24 h cells in the lower chamber were counted. The experiment was repeated on four occasions. The neural progenitor cells (NPCs) from three different mice of each genotype were used on each occasion, and the experiment on cells from each mouse was repeated three times.

### Quantification and statistical analysis

BrdU-positive cells were counted (double-blinded) and scored using Olympus CAST-Grid stereology and Fractionator method. Statistical significance was determined by one-way anova/Neumann–Kuels *post hoc* test.

## Results

### OBs of prokr2^Brdm1^ homozygous mice are smaller and disorganized

**Fig. 1:**
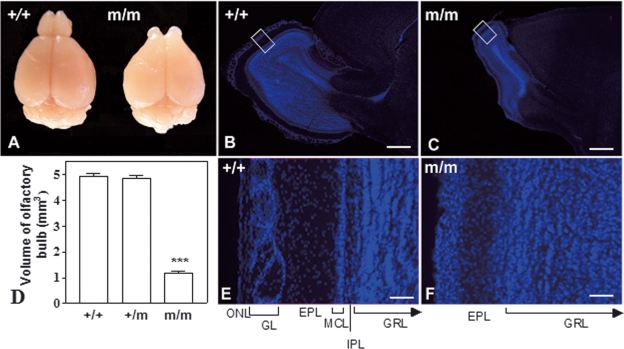
Analysis of OBs. Macro- and microscopic analysis of OB from *m/m* and +/+ mice. (A) Macroscopic view of a *m/m* mouse compared with a +/+ littermate at 9 weeks old. (B) +/+ OB showing normal size of OB. (C) *m/m* OB showing decreased size compared with +/+. (D) *m/m* show decreased volume of the OB compared with either +/+ or +/*m* (***P < 0.001 versus +/+, +/*m*; *n* = 6). (E) Boxed region in (B) and (F), boxed region in (C), comparison of architecture of OB in (B), +/+ vs (C), *m/m* mice. There is a decrease in the overall size of the OB, which can be contributed to a complete absence of a discernable olfactory nerve layer (ONL), glomerular layer (GL), mitral cell layer (MCL) and internal plexiform layer (IPL). There is evidence of the external plexiform layer (EPL) in the homozygous and also the granule cell layer (GRL). Scale bars: 1 mm (B and C); 200 µm (D and E). Arrows in (E) and (F) indicate that the GRL extends beyond the edge of the figure.

**Fig. 2:**
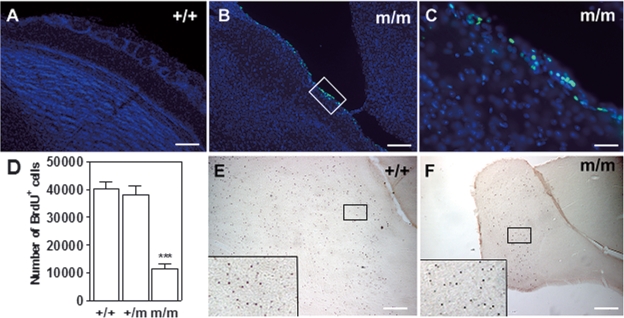
TUNEL staining and cell proliferation in the OB. (A) *+/+* OB showing lack of TUNEL staining compared with (B) TUNEL-positive staining in the *m/m* mice shown in higher-power magnification in (C) Note, staining is highest in the GL where architecture is distorted (see also Fig. 1). (D) There is a decrease in bromodeoxyuridine (BrdU) staining in the OB of *m/m* mice compared with +/+ or +/*m* 21 days after a single injection of BrdU (***P < 0.001 versus +/+, +/*m*; *n* = 6). (E) BrdU staining of OB in +/+ mice (magnification 5 ×). The boxed region in (E) is shown in the corner of (E). (F) BrdU staining in OB of *m/m* mice (magnification 5 ×), the boxed region in (F) is shown in the corner of (F). Note, the difference in size of the OB at 5 × magnification. Scale bars: 100 µm (A and B); 20 µm (C); 500 µm (E and F).

The generation of the *prokr2*^*Brdm1*^ (abbreviated here to *m*) allele has been described elsewhere (Prosser *et al*., 2007). Anatomical analysis of the brain demonstrated that the sizes of the right and left OB were symmetrically reduced in *m/m* mice (*n* = 7) when compared with their +/+ littermate controls (*n* = 6; Fig. 1A–D) and +/*m* controls (*n* = 7; data not shown). In addition, the normal morphology of the OB was distorted, in that there was a distinct absence of the glomerular layer (GL), mitral cell layer and internal plexiform layer in *m/m* mice (Fig. 1E and F). Volumetric analysis of the *m/m* OB demonstrated that they are four times smaller than those of either +/+ or +/*m* littermates (Fig. 1D). In addition, the width of the OB was narrower in *m*/*m* mice than either their +/+ or +/*m* littermates (+/+ 870 + 33; +/*m* 880 + 40; *m/m* 395 + 20 µm). In order to determine if ongoing cell death contributes to the OB defect of the *m/m* mice, TUNEL staining was performed that revealed this was indeed the case (Fig. 2A–C). Interestingly, TUNEL-positive nuclei were most pronounced in the area that should constitute the missing GL (Fig. 2C). There was no difference in TUNEL-positive staining between *m/m* and control mice elsewhere in the brain (data not shown).

### Newborn prokr2^Brdm1^ homozygous C57BL/6 inbred mice suckle effectively

As previously documented, the *m/m* mice exhibit partially penetrant postnatal lethality during the first few days after birth (Prosser *et al*., 2007). The penetrance of the lethality was very severe upon backcross to the C57BL/6 genetic background. Despite this, P0 *m/m* mice of the N8 C57BL/6 backcross generation showed an obvious milk band comparable in volume to their +/*m* and +/+ littermates (Supplementary material, Fig. S1). Therefore, deficiency in the ability to suckle and compete with littermates is not apparent during the first day after birth.

### Exclusive loss of NPCs in the OB of Prokr2 null mice

In order to determine if the difference in OB size might be accounted for by a difference in the numbers of dividing progenitors, BrdU incorporation was counted 3 weeks after a single BrdU injection. Results demonstrate that there was indeed about a threefold decrease in the OB BrdU-positive cells of *m/m* mice compared with +/*m* or +/+ littermates (Fig. 2D–F). In addition, short-term BrdU experiments where animals were killed 24 h after five daily BrdU injections also showed a decrease in labelling in the OB in *m/m* mice (+/+ 102 071.5 ± 5499; *m/m* 25 283 ± 1649; +/*m* 98 989 ± 9336). However, there was no difference in cell numbers in the SVZ (+/+ 1844 ± 652; *m/m* 1572 ± 363; +/*m* 1765 ± 412), indicating that cell proliferation is not defective but their arrival to the OB is impeded. The BrdU-positive cells are being delayed in the RMS (data not shown).

In order to determine that the lack of difference in SVZ proliferation might be due to a survival effect over the 5 days of BrdU administration, we gave a single injection of BrdU and killed the animals 2 h later. Results show that there was no difference in the number of BrdU-positive cells in the SVZ at this time (+/+ 1903 + 261; *m/m* 1750.61 + 259; +/*m* 1560.05 + 213), again indicating that cell proliferation is not defective.

We next examined if proliferation in another neurogenic region, the dentate gyrus, was affected. Results demonstrate that there was no difference in proliferation between *m/m* and +/+ or +/*m* mice in this region (+/+ 1003 ± 344; *m/m* 1237 ± 39; +/*m* 1168 ± 165). In all cases the counts for BrdU-positive cells were not significantly different between +/*m* and +/+ mice.

### Decreased TH-positive neurons in the OB of Prokr2 null mice

**Fig. 3:**
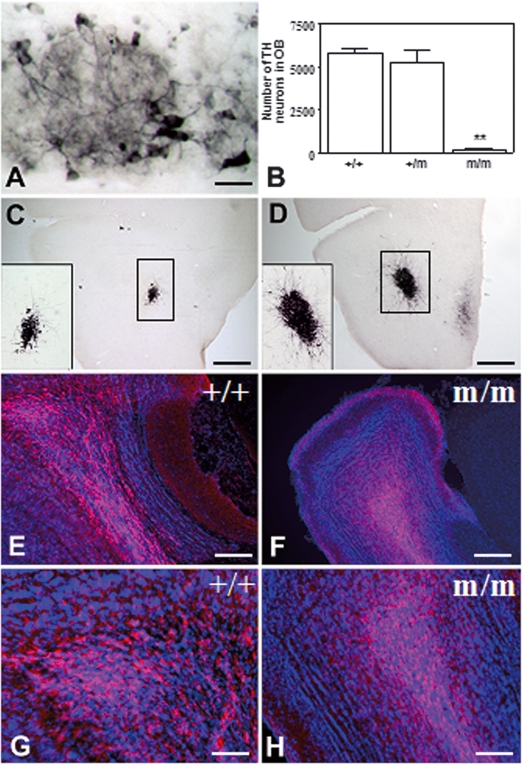
Neuronal differentiation in olfactory bulb (OB) and RMS. (A) Tyrosine hydroxylase (TH) staining in the GL of the +/+ OB. (B) There is severe depletion of TH in the OB GL of *m/m* mice compared with either +/+ or +/– (**P < 0.01 versus +/+, +/*m*; *n* = 5). (C) DCX-positive neurons in the RMS of +/+. (C′) 20 × magnification. (D) DCX-positive neurons in the RMS of a *m/m* mouse. (D′) 20 × magnification. Note the greater number of DCX-positive neurons in the RMS of *m/m* mice, indicating their retention there compared with +/+. (E) PSA-NCAM staining in the olfactory ventricle of +/+, compared with (F), *m/m* mouse. (G) Higher magnification (20 ×) of (E), (H) higher magnification (20 ×) of (F), demonstrating that PSA-NCAM appears to be in tighter bundles in the *m/m* compared with +/+ littermates. Scale bars: 10 µm (A); 1 mm (C and D); 250 µm (E and F); 125 µm (G and H).

Given that there is abnormal architecture of the OB, we next determined if genesis of dopaminergic neurons was affected. TH is a marker for dopaminergic neurons normally present within the GL of the OB. We report here that the number of dopaminergic neurons in this region is decreased in *m/m* mice (Fig. 3A and B). Further examination of the OB revealed that calbindin-positive neurons were found predominately in the GL of the +/+ mice. In contrast, this population of neurons was dispersed throughout the OB of the *m/m* mice, indicating that their differentiation in their final destination (GL) is impeded (supplementary Fig. S2). Examination of the RMS revealed that there was an increase in newborn neurons that express DCX in *m/m* mice (Fig. 3C and D). DCX-positive neurons occupied 8.34 ± 1.19% of the area of the RMS in *m/m* mice, whereas they occupied 1.29 ± 0.25% in +/+ mice, signifying a defect in their ability to reach the OB in *m/m* mice. In addition, PSA-NCAM-labelled neuronal progenitors are increased in the olfactory ventricle of *m/m* mice with progenitors appearing in tighter clusters relative to control mice, indicating a failure in disassociation from their homotypic chains (Fig. 3E–H).

### SVZ progenitors of Prokr2 null mice lose their ability to migrate towards its ligand

**Fig. 4:**
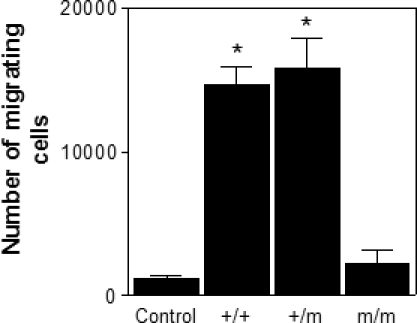
Transwell assay. There is a decrease in migration of cells grown from *m/m* mice towards the Prokr2 receptor ligand PROK2 compared with +/+ or +/*m* littermates (*P < 0.05 versus Control + *m*/*m*, +/*m*). Control wells contained no PROK2 and demonstrated limited migration over the 24-h period.

In order to determine the effect of PROKR2 loss upon Prok2 chemoattraction for SVZ progenitor cells, we used a transwell assay. NPCs derived from +/+, +/*m* and *m/m* mice at P0 were expanded in EGF and FGF2 for 4 days, at the end of which they were dissociated to a single cell suspension and plated in the upper chamber. They were allowed to migrate through the membrane filter separating the upper and lower chambers towards a source of recombinant *Prok2*. Results show that *m/m* cells had a decreased ability to migrate compared with either +/+ or +/*m* cells (Fig. 4). The migration of *m/m* cells was not significantly different from that of the negative control, namely treatment of +/+ cells with media containing no PROK2 (Fig. 4).

## Discussion

The persistence of new neuronal production within the OB throughout the lifetime of an animal suggests this process has a fundamental biological significance. This study has directly addressed the importance of Prokr2 signalling in this process, by generating a null mutation. One of the most striking effects in the CNS of the Prokr2 null mice was a marked decrease in the size of the OB, as has been previously described (Matsumoto *et al*., 2006). In addition, the layering of the OB was abnormal, the six well-described layers: nerve, glomerular, external plexiform, mitral, internal plexiform and granular were not all present. In fact, the glomerular, mitral and internal plexiform layers were all anatomically indiscernible. In addition to distorted morphology, there is also a significant decrease in the A16 group of dopamine neurons in Prokr2 null mice, as evidenced by a significant reduction in TH-positive neurons in the periglomerular layer. Indeed, it has previously been shown that TH is not expressed on these cells until they reach their final destination (McLean & Shipley, 1988). Interestingly, the location of another population of calbindin-positive interneurons, that are preferentially found in the GL, with a smaller proportion in the granule cell layer (Hwang *et al*., 2002) was distorted in the Prokr2 null mice. In these mice the neurons appeared relatively immature and were found throughout the OB and not concentrated in the GL as in their wild-type littermates. Therefore, their reduced numbers may be due to fewer reaching their final destination, combined with evidence of TUNEL-positive apoptotic nuclei in the vicinity of this distorted layer. It is plausible that both phenomena contribute to a reduction in these interneurons in this region. Indeed, Prok2 has been shown to protect cells derived from the corpus luteum against apoptotic cell death due to serum starvation (Kisliouk *et al*., 2005).

A marked deficiency in OB development has previously been reported in the Prokr2 null mice as early as E16.5 (Matsumoto *et al*., 2006) and P1 (M.A.C., unpublished observations), suggesting that Prokr2 is critical for the normal development of the OB. It is of interest that in *Prok2* knockout mice asymmetric OB malformation was reported in approximately 50% of the mice (Ng *et al*., 2005), whereas we and others (Matsumoto *et al*., 2006) found symmetrical OB malformation in all of our Prokr2 null mice. Moreover, the degree of organization in the OB of Prok2 null mice appears to vary with the mitral cell layers being discernable in some but absent from other OB lobes (Ng *et al*., 2005; Zhang *et al*., 2007) These observations suggest that the Prokr2 null OB phenotype is more extensive than that for its ligand Prok2 and confirms the critical role of this receptor for OB neurogenesis. In contrast, the more organized structures of the OB in Prok2 nulls suggest that loss of the ligand can be partially compensated by alternative mechanisms. However, the lack of *Prok1* mRNA expression detected in the OB (Cheng *et al*., 2006) raises doubt over whether it can functionally substitute for Prok2 in the null mutant.

Tbrain-1 (Tbr-1) and the lysophosphatidic acid receptor (LpA1) null mutant phenotypes, although mechanistically different, do share some of the features of defective OB with the Prokr2 phenotype described here. Null mutants for the Brachyuri homologue *Tbr*-*1* are deficient in OB mitral and tufted cells (Bulfone *et al*., 1998), whereas the *LpA1* null mice exhibit a probable deficit in neuronal progenitor proliferation and a partially penetrant reduction in OB wall thickness (Contos *et al*., 2000). It is of interest that null mutants for *Tbr-1* and *LpA1* with deformed OB are deficient in suckling ability contributing to postnatal lethality. Similarly, the Prok2 and Prokr2 nulls exhibit partial penetrant lethality that might be caused by olfactory deficits compromising suckling (Li *et al*., 2006; Matsumoto *et al*., 2006; Prosser *et al*., 2007). However, even in C57BL/6 backcrossed mice where the penetrance of postnatal lethality is almost complete, Prokr2 null mice do have milk in their stomachs. This suggests that olfactory impairment of suckling ability is unlikely to be the critical factor in Prokr2 null postnatal lethality.

Cell division in the SVZ is responsible for new neurons being added to the granular and the periglomerular layers of the OB. These cells migrate through the RMS, where the majority disperse throughout the granule layer and a small percentage develop into interneurons in the periglomerular layer. However, in the Prokr2 null mice the volume of the OB is approximately fourfold less than wild-type littermates, and the cell numbers reaching the OB are reduced by about threefold. This is not due to a decrease in cell division in the SVZ as no deficiency was noted in Prokr2 nulls. Instead it is their migration that is compromised as they are being retained within the RMS and fail to migrate from there out into the granular layer of the OB. This results in a deficit in differentiated granule cells and is likely to be responsible for the marked reduction in the size of the OB and its distorted architecture. This would suggest that the lack of Prokr2 might abolish Prok2 signalling, which acts as a detachment signal for chain migrating progenitors arriving from the RMS (Ng *et al*., 2005). Further support for this comes from the increased accumulation and tighter clustering of PSA-NCAM-positive neuronal progenitors around the olfactory ventricle of Prokr2 null mice relative to wild-type controls. In addition, DCX-positive neuronal progenitors accumulated in the RMS, representing a higher percentage of cells than in wild-type littermates. The evidence we present here that Prokr2 activation is essential for the migration and differentiation of neuronal progenitors is similar to the OB phenotype of Mash1 null mutants (Murray *et al*., 2003). Interestingly, it has recently been reported that the expression of Mash1 and neurogenin1 (Ngn1) overlap with Prok2 in several neuronal populations of the OB, and that Prok2 is decreased by 70% in Ngn1 null mice at P0 and 50% in Mash1 null mice at E16.5 compared with their respective wild-type littermates (Zhang *et al*., 2007). In addition, both of these basic-helix-loop-helix transcription factors are proposed to regulate Prok2 expression, and ChiP assays have shown binding to the Prok2 promoter E-box motifs (Zhang *et al*., 2007). This would suggest that Prok2 may mediate a functional role for both Mash1 and Ngn1 in OB neurogenesis.

Quantitative evidence for the critical role of Prokr2 and the lack of redundancy in the prokineticin receptors in mediating chemoattraction by prokineticins was provided by transwell migration experiments where SVZ cells from Prokr2 null mice demonstrated insignificant PROK2-dependent migration relative to wild-type explants in the absence of chemoattractant. This supports the morphological examination of Matsumoto *et al*. (2006) showing that exclusively Prokr2 and not Prokr1 knockouts display an OB phenotype.

The phenotypes of *Prokr2* and *Prok2* knockout mice model aspects of human Kallman syndrome (KS), a partially penetrant genetic disease that causes OB deformity and consequently anosomia and hypogonadism with associated sterility and failure to reach puberty (Ng *et al*., 2005; Matsumoto *et al*., 2006). It is likely that the reproductive disorders are caused primarily by the absence of neurons producing gonadotrophin-releasing hormone (Schwanzel-Fukuda *et al*., 1989), as is the case in the Prokr2 null mouse (Matsumoto *et al*., 2006). Approximately 20% of KS cases are caused by loss of function mutations in either the X-linked *KAL1* gene, that encodes anosmin-1, or the fibroblast growth factor receptor 1 gene (*FGFR1*), responsible for an autosomal dominant form of the disease. It was recently established that mutations within *PROK2* (heterozygous) and *PROKR2*(homozygous, heterozygous or compound heterozygous) are present within 10% of a cohort of patients with KS (Dode *et al*., 2006). Thus, a deficiency in PROKR2 signalling is a likely cause of KS in some patients. The presence of heterozygous mutations within the *PROKR2* and *PROK2* genes of patients with KS suggests that either partial PROKR2 signalling impairment is sufficient to cause the KS on its own or that inheritance of the disorder is dependent upon mutations in additional genes involved in the same, or convergent, signalling pathways (Dode *et al*., 2006).

In conclusion, we show that normal functioning of Prokr2 is necessary for the migration of homotypic chains of neuroblasts through the RMS and their detachment prior to their arrival at their final destination in either the granule or GL of the OB. Through this mechanism this G-protein-coupled receptor is crucial for establishment of the normal layered structure and ongoing neurogenesis of the OB.

## Abbreviations

BrdU: bromodeoxyuridine
DAB: diaminobenzidine
DCX: doublecortin
DMEM: Dulbecco's modified essential medium
EGF: epidermal growth factor
FGF: fibroblast growth factor
GL: glomerular layer
KS: Kallman syndrome
NPC: neural progenitor cell
OB: olfactory bulb
Prok: prokineticin
Prokr: prokineticin receptor
PSA-NCAM: polysialylated form of the neural cell adhesion molecule
RMS: rostral migratory stream
SVZ: subventricular zone
TBS: Tris-buffered saline
TH: tyrosine hydroxylase
TUNEL: terminal dUTP nick-end labeling.

## Supplementary material

The following supplementary material may be found on http://www.blackwell-synergy.com

Fig. S1 Prokr2 *m*/*m* mice are able to suckle milk.

Fig. S2 Calbindin-positive neurons in the OB.
