# Supplementary material for: Olfactory bulb hypoplasia in Prokr2 null mice stems from defective neuronal progenitor migration and differentiation
Source: Eur J Neurosci. 2007 Dec;26(12):3339–44. doi: 10.1111/j.1460-9568.2007.05958.x (PMC2228368; doi:10.1111/j.1460-9568.2007.05958.x)
Supplement: Fig. S1 — Prokr2 m/m mice are able to suckle milk. [file ejn0026-3339-SD1.doc]

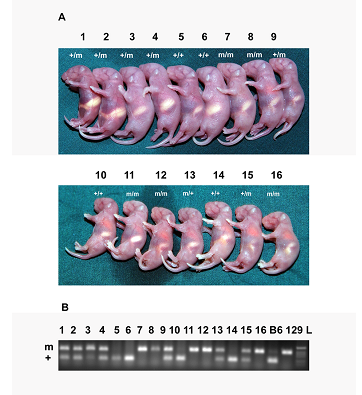


Fig. S1. Prokr2 *m*/*m* mice are able to suckle milk. A, P0 mice from two litters of N8F1 C57BL/6 backcrossed generation were photographed to show the presence and size of the milk band. B, the mice were subsequently genotyped by PCR as previously described (Prosser et al, 2007) The 373bp *Prokr2* intron 2 PCR fragment is restriction digested using AluI to release 176bp and 197bp fragments in the C57BL/6 (+) alleles but not 129 (*m*) allele. B6: C57BL/6 control DNA. 129: 129 control DNA. L: 1kb DNA ladder (Invitrogen, Paisley, UK); upper fragment is the 506/517bp doublet.
