# Supplementary material for: Olfactory bulb hypoplasia in Prokr2 null mice stems from defective neuronal progenitor migration and differentiation
Source: Eur J Neurosci. 2007 Dec;26(12):3339–44. doi: 10.1111/j.1460-9568.2007.05958.x (PMC2228368; doi:10.1111/j.1460-9568.2007.05958.x)
Supplement: Fig. S2 — Calbindin-positive neurons in the OB. [file ejn0026-3339-SD2.doc]

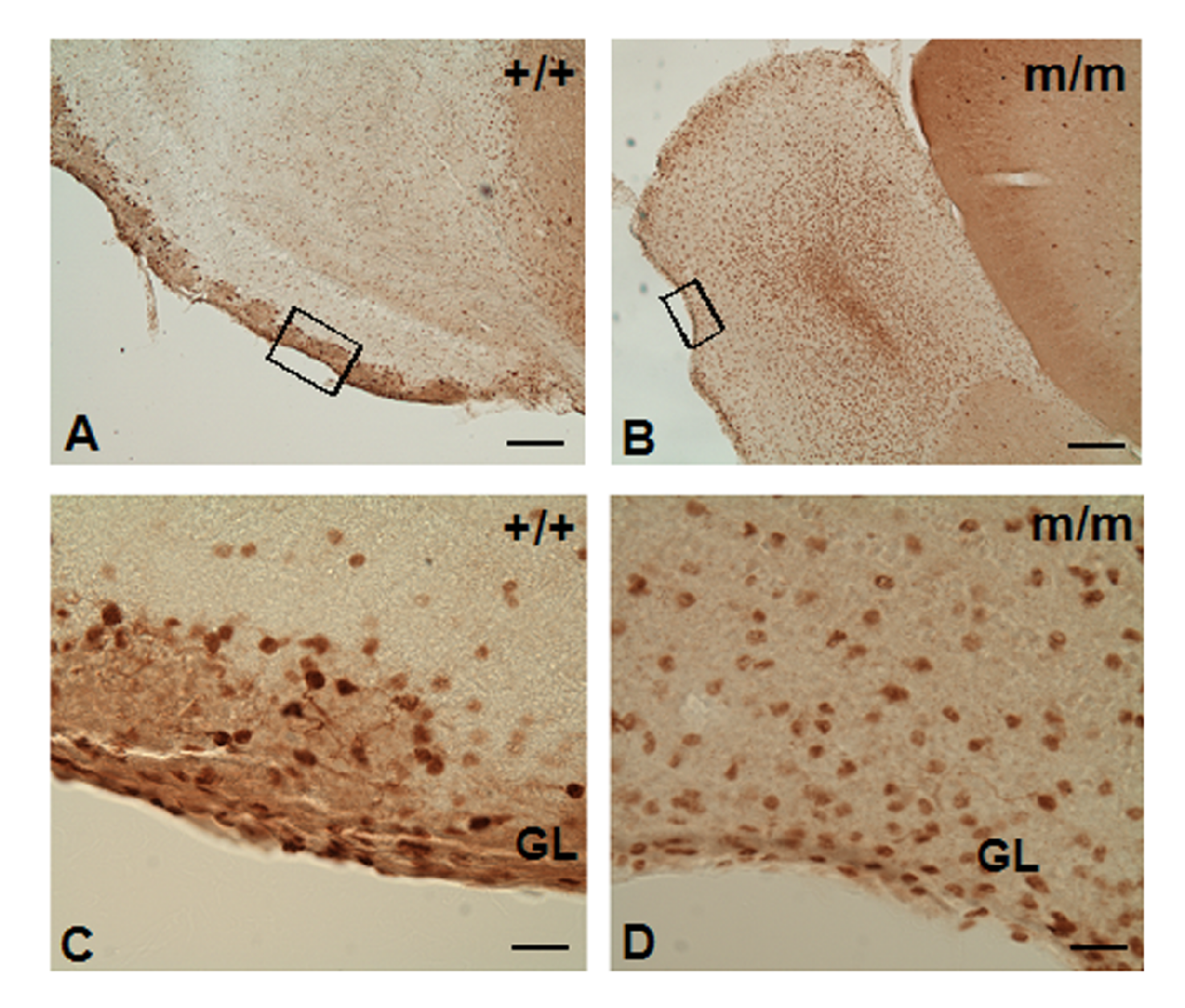


Fig. S2. Calbindin positive neurons in the OB. A, calbindin staining in the OB of +/+ mice. B, calbindin staining in the OB of m/m littermates. C, boxed region of A, indicating a concentration of positive neurons in the glomerular layer. D, boxed region of B, indicating that the staining is not concentrated in the glomerular layer and is dispersed throughout the other layers of the OB. GL is glomerular layer. Scale bars A,B, 250 µm, C,D, 25 µm.
